# CTCs-oriented adjuvant personalized cytostatic therapy non-metastatic breast cancer patients: continuous non-randomized prospective study and prospective randomized controlled study

**DOI:** 10.1007/s10549-020-06036-z

**Published:** 2021-01-03

**Authors:** Ya A. Shliakhtunou

**Affiliations:** Department of Oncology, Educational Establishment “Vitebsk State Medical University”, Frunze Av., 27, 210009 Vitebsk, Republic of Belarus

**Keywords:** Breast cancer, Circulating tumor cells, Adjuvant chemotherapy

## Abstract

**The aim:**

To conduct a prospective randomized controlled study of the optimization of adjuvant therapy in patients with non-metastatic breast cancer, taking into account the presence of circulating tumor cells (CTCs) with an assessment of tumor-specific OS and DFS.

**Materials:**

*Stage 1* Continuous non-randomized prospective study (*n* = 102) to study the clinical and prognostic value of CTCs and evaluate the effectiveness of adjuvant systemic therapy in relation to CTC eradication; *Stage 2* Prospective randomized controlled study (*n* = 128) of optimization of adjuvant therapy taking into account CTCs with an assessment of the effectiveness of the standard therapy and an optimized therapy regimen.

**Results:**

Monitoring of CTCs during adjuvant drug treatment has established that a significant decrease in the frequency of CTC identification can be achieved only by sequential administration of anthracyclines and taxanes (paclitaxel) AC-T, which allows reducing CTCs compared to other regimens from 52.6 to 15.8% (*p* = 0.006).

CTC-oriented personalized adjuvant therapy in the experimental group, based on the timely transition from an ineffective adjuvant chemotherapy regimen to taxanes, as well as additional monochemotherapy with gemcitabine can achieve 100% eradication CTCs. In the adjuvant therapy experimental group taking into account CTCs (*n* = 68), the OS 5-year tumor-specific rate was 90.3 ± 3.8%, (control group 78.7 ± 3.9%, *p* = 0.036). DFS tumor-specific in the experimental group was 88.0 ± 4.4%, (control group 80.6 ± 3.3%, *p* = 0.023).

**Conclusions:**

The use of the method of treatment of CTC-oriented personalized adjuvant therapy for non-metastatic breast cancer makes it possible to reliably increase DFS 5-year by 7.4% and OS 5-year by 11.6%.

## Introduction

The potential role of CTCs and DTCs (together with therapy-resistant cancer cells, collectively referred to as MRDs) in clinical practice is gradually moving toward the possibility of using them in the framework of normal routine practice. CTCs, in particular, realize the exciting prospect of "liquid biopsy" because blood sampling is minimally invasive and very fast, allowing access to proliferating cancer cells. In any case, the idea that these cells can serve as an important source of genetic information in disseminated disease and be regularly monitored during cancer treatment will allow for continuous, modern, planned, optimized, and personalized treatment [1]. At the same time, the presence of MRD, as well as its persistence during systemic therapy, is associated with a worse prognosis. To date, as estimated by ASCO and SWOG S0500 [2, 3], there is insufficient evidence of clinical utility to support routine MRD research during routine clinical practice. A pilot study of CirCe-01 in France is ongoing, which also showed a strong predictive value for CTC counts as a baseline before treatment [3]. It is clear that CTC and DTC can differ from each other both among patients in general and within the same patient, and this explains why variable results are observed in patients positive for CTC and DTC, which in turn was a key question of the group. the ASCO consensus [2].

Thus, advancing knowledge of the biology of these cells has become a central focus and key to their application. Despite the fact that there are great technical problems in this area of research, within a fairly short period of time, much has become known about MRD in terms of such aspects as heterogeneity, inconsistency with the primary tumor and metastases, EMT and tumor stem cells, resistance to therapy, survival, behavior with neighboring cells and microenvironment, and most importantly, changes in our understanding of the nature of metastasis. The results achieved in the study of CTCs and DTCs over the past two decades are unprecedented (Medline has only over 20,000 publications on CTCs). The predictive relationship between the presence/count of CTCs is undeniable, and new horizons for diagnosis and therapeutic targeting are compelling [4–6]. At the same time, most of the data were obtained as a result of experimental data, in the course of studying various models of tumor progression, and we can only assume that such processes occur in a human body suffering from a malignant tumor. Therefore, clinical prospective randomized trials are fundamental for the use of CTCs as prognostic and predictive markers for the treatment of malignant tumors, including breast cancer.

The aim of the study is to conduct a prospective randomized controlled study of the optimization of adjuvant therapy in patients with non-metastatic breast cancer, taking into account the presence of circulating tumor cells in the peripheral blood (minimal residual disease) with an assessment of tumor-specific overall and relapse-free survival.

## Materials and methods

The research consisted of several stages.

- *Stage 1* Continuous non-randomized prospective study (*n*  = 102) to study the clinical and prognostic value of CTCs, as well as to study and evaluate the effectiveness of adjuvant systemic therapy in patients with non-metastatic breast cancer in relation to CTC eradication;
- *Stage 2* Prospective randomized controlled study (*n*  = 68 experimental group, *n*  = 60 control group) of optimization of adjuvant therapy in patients with non-metastatic breast cancer taking into account CTCs with an assessment of the effectiveness of the standard therapy regimen and an optimized therapy regimen with an assessment of tumor-specific overall and disease-free survival.

Randomization was carried out according to the principle of adaptive randomization using the Maximum Utility Model, when the next patient is always assigned to the group in which there is (or is assumed based on the model) a higher treatment efficiency. Randomization was performed after morphological confirmation of the diagnosis prior to initiation of specific antitumor treatment.

### Study inclusion criteria

- Age over 18;
- Cytological confirmation of breast cancer diagnosis;
- Histological confirmation of the diagnosis of breast cancer with the determination of the expression of receptors for estrogens and progestins, as well as the expression / amplification of the epidermal growth factor receptor HER2-neu and the index of proliferative activity Ki 67;
- Nodular breast cancer;
- Unilateral defeat;
- Stage pT1-4N0-3bM0 according to TNM;
- Resectability of the tumor in the amount of radical Madden mastectomy or radical resection of the breast;
- Operability of patients (status on the ECOG scale from 0 to 2 points);
- Absence of other synchronous and metachronous malignant tumors, including in the anamnesis;
- Absence of pregnancy;
- The patient's ability to follow the doctor's recommendations and follow the study design.

### Patient exclusion criteria

- Unwillingness of the patient to continue participating in the study;
- Serious adverse events experienced by patients during the study;
- Individual intolerance to drugs included in the therapy regimen during the study;
- The presence of a second malignant tumor;
- The presence of contraindications to the appointment of antitumor chemotherapy: symptoms of congestive cardiovascular failure, early periods after myocardial infarction (up to 6 months), unstable exertional angina, uncontrolled cardiomyopathy, arrhythmias, arterial hypertension, early periods after a stroke.

### Characteristics of patients of the 1st stage of the study

The average age of patients was (M ± SD) 58.0 ± 12.7 with individual fluctuations from 31 to 91 years.

The incidence of damage to both left and right mammary glands was practically the same, 50.5% and 49.5%, respectively.

Most often, the tumor node was localized in the upper outer quadrant (61.9%).

Tumors of the T1 and T2 categories were found predominantly; up to 5 cm in the largest size (53.9% and 43.1%, respectively). In 48.0% of cases, regional lymph nodes remained not involved in the tumor process. In 39.2% of cases, there was a metastatic lesion of the I lymph nodes in the amount from 1 to 3. Greater lesions of the regional lymphatic collector corresponding to the N2–N3 category were less common (3.9% and 8.8%, respectively). The overwhelming majority of patients in the experimental subgroup were women with breast cancer stages I and IIA (66.6%), the share of locally advanced forms IIB–IIIC was 33.4%.

According to the data of pathomorphological examination, invasive unspecified (ductal) carcinoma was diagnosed most often, in 74.5% of cases, lobular carcinoma was diagnosed more than three times less often (21.6%). Other types of cancer (tubular, medular, mucinous carcinomas) were significantly less common and totaled 3.9%. The tumor was usually high (G3) or medium (G2) grade (52.9% and 44.1%, respectively) and had lymphovenous stromal invasion (LVSI +  − 84.3%). Luminal A cancer was identified in 48.0% of cases, non-expressing luminal B HER2—in 25.5%, expressing luminal B HER2—in 5.9%, HER2 overexpressing—in 6.9%, and three times negative subtype were diagnosed in 13, 7% of cases.

Clinical-anatomical, pathological-morphological, and molecular biological characteristics of the primary tumor are presented in Table 1.

**Table 1:** Clinical-anatomical, pathological-morphological, and molecular biological characteristics of the tumor of patients of the 1st stage of the continuous non-randomized prospective study

Tumor characteristic	*n* = 102	
	Abs. values	%
*T*		
1	55	53.9
2	44	43.1
3	2	2.0
4	1	1.0
*N*		
0	49	48.0
1	40	39.2
2	4	3.9
3	9	8.8
Stage		
I	34	33.3
IIA	34	33.3
IIB	18	17.6
IIIA	5	4.9
IIIB	1	1.0
IIIC	10	9.8
Histological structure of carcinoma		
Tubular	1	1.0
Medular	1	1.0
Mucinous	2	2.0
Unspecified	76	74.5
Lobular	22	21.6
Greiding		
G1	3	2.9
G2	45	44.1
G3	54	52.9
LVSI		
LVSI +	86	84.3
LVSI–	16	15.7
Molecular biological subtype of tumors		
Luminal A	49	48.0
Luminal B HER2–	26	25.5
Luminal B HER2 +	6	5.9
Overexpressing HER2	7	6.9
Thriple negative	14	13.7

The characteristics of the tumor process of patients with breast cancer included in the randomized controlled trial are presented in Table 2.

**Table 2:** Patient characteristics of a randomized controlled trial

Tumor characteristic	Control group *n* = 68		Experimental group *n* = 68	
	Abs. values	%	Abs. values	%
*T*				
1	32	53.3	34	50.0
2	26	43.3	30	44.1
3	1	1.7	4	5.9
4	1	1.7	0	0.0
*N*				
0	29	48.3	30	44.1
1	24	40.0	24	35.3
2	2	3.3	4	5.9
3	5	8.3	8	11.8
Stage				
I	20	33.3	24	35.3
IIA	20	33.3	20	29.4
IIB	11	18.3	14	20.6
IIIA	3	5.0	4	5.9
IIIB	1	1.7	1	1.5
IIIC	6	10.0	5	7.4
Histological structure of carcinoma				
Tubular	1	1.7	1	1.5
Medular	0.6	1.0	1	1.5
Mucinous	1.2	2.0	1	1.5
Unspecified	44.7	74.5	50	73.5
Lobular	12.96	21.6	15	22.1
Greiding				
G1	1.74	2.9	1	1.5
G2	26.46	44.1	27	39.7
G3	31.74	52.9	40	58.8
LVSI				
LVSI +	50.58	84.3	57	83.8
LVSI–	9.42	15.7	11	16.2
Molecular biological subtype tumors				
Luminal A	28.8	48.0	30	44.1
Luminal B HER2–	15.3	25.5	18	26.5
Luminal B HER2 +	3.54	5.9	5	7.4
Overexpressing HER2	4.14	6.9	4	5.9
Thriple negative	8.22	13.7	11	16.2

### Statistics

Statistical processing of the data obtained was carried out in accordance with modern requirements for medical and biological research.

Qualitative indicators are presented in absolute and relative values.

The distribution of quantitative traits for normality was checked using the Lilliefors and Shapiro–Wilk criteria. Quantitative features obeying the normal distribution law are presented as mean value (M), standard deviation (SD), standard error of the mean (SE), and minimum and maximum values (min, max).

Quantitative signs that do not obey the normal distribution law are shown as a median (Me), interquartile range (LQ/UQ), and minimum and maximum values (min, max).

Comparison of the two groups in terms of quantitative characteristics having equal general variances and corresponding to the normal distribution law was carried out using the Student's test. The condition of equality was checked according to the criteria of Leuven and Fisher. Comparison of the two groups in terms of quantitative characteristics that do not correspond to the normal distribution law was carried out using the non-parametric Mann–Whitney test. Comparison of groups by qualitative ordinal characteristics was carried out according to the Mann–Whitney test, qualitative nominal—by the Pearson chi-square (*χ*2) test, by qualitative binary—by the *χ*2 test with Yates' correction and Fisher's exact test in accordance with the conditions of their applicability.

Pearson's correlation coefficient was calculated as a measure of the relationship for quantitative traits that obey the normal distribution law. For quantitative traits that do not obey the normal distribution law, and qualitative ordinal traits—Spearman's rank correlation coefficient, in some cases—the Mann–Whitney coefficient.

To determine the degree of heterogeneity, cluster analysis was used with the construction of a hierarchical tree (tree diagram) with an estimate of the Euclidean connection distance. Also, the cluster analysis used the two-input combining method.

To assess the long-term results of treatment, the values of overall and disease-free survival were calculated using the Kaplan–Meier method. Comparison of survival in the two groups was carried out using the log-rank test.

Comparison of groups by long-term results of treatment was also carried out in terms of the relative risk of death from any cause and the relative risk of recurrence and progression of the disease. Risk ratios (RRs), 95% confidence intervals for the risk ratios and the significance level of various risks were calculated. The relative risk and its 95% confidence interval were calculated using the Cox proportional hazards regression model.

To identify indicators that affect the risk of recurrence and progression of the disease, a monovariant analysis was carried out for all individual indicators. Risk-related indicators with a statistical significance level of *p* < 0.05 are included as predictors in the multivariate model.

In all cases, the differences were considered statistically significant at a significance level of *p* < 0.05. All *p* values were two-sided. Statistical processing of the results was performed using the SPSS Statistics 10.0 software.

### Enrichment, isolation, and identification of circulating tumor cells

At the stages of treatment, a 5 ml sample of peripheral blood from the cubital vein in the morning on an empty stomach was taken from all patients in a sterile vacuum tube with K2EDTA for subsequent enrichment and isolation of CTCs and stored at 4 °C until the study. Samples were processed immediately or no later than four hours after blood collection.

The enrichment and isolation of CTCs was carried out using the technology of rapid isolation of tumor cells from whole blood based on covalently bound antibodies for CD326 on a non-magnetic polymer matrix of large microspheres with subsequent isolation of CTCs by size (S-pluriBead Maxi Reagent Kit and anti-human CD326 S- pluriBead, Germany) with subsequent identification by expression of the Survivin (BIRC5) and HER2-neu (SIVital, Belarus) genes using Real-time PCR.

## Results

In the study of peripheral blood of patients in the prospective study group of standard treatment (*n* = 102) with verified primary non-metastatic breast cancer in the morning before surgery, the expression of at least one marker gene in the CTC was detected in 69 women out of 102 examined, which amounted to 67.6%.

After radical surgery, the frequency of CTC-positive patients was 45 (44.1%).

After the completion of all therapy, the detection rate of CTC-positive patients was 41 (40.2%).

In the prospective study group (*n* = 102), the overall five-year tumor-specific survival rate was 86% ± 3.7%, the relapse-free tumor-specific survival rate was 84.4% ± 4.0%. A summary is presented in Table 3.

**Table 3:** Survival of patients in the continuous non-randomized prospective study group

Survival	Survival rates, % ± SE		
	1 year old	3 year old	5 year old
Overall survival	97.6 ± 1.5%	88.7 ± 3.1%	86.0 ± 3.7%
Disease-free survival	94.1 ± 2.3%	86.9 ± 3.4%	84.4 ± 4.0%

**Fig. 1:**
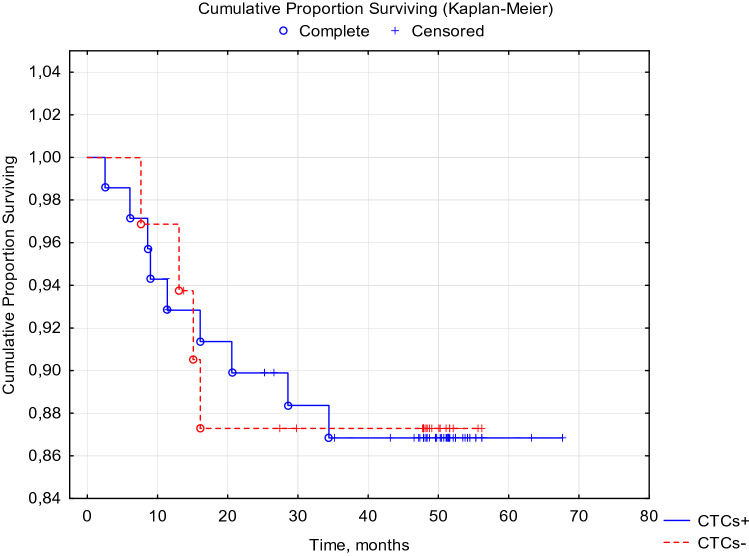
DFS of breast cancer patients with BIRC5- and HER2-neu mRNA-positive CTCs before treatment. Log-Rank Test WW = 0.06526, Sum = 12.839, Var = 2.7916, Test statistic = 0.0390571, *p* = 0.96884

**Fig. 2:**
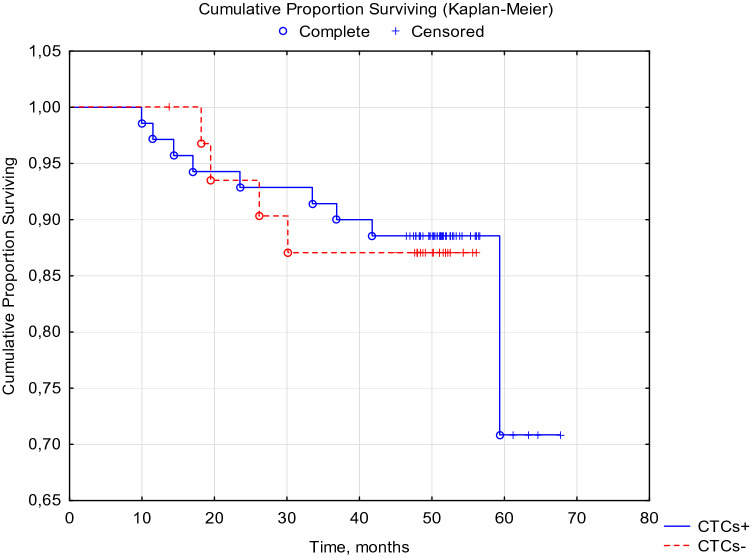
OS of breast cancer patients with BIRC5- and HER2-neu mRNA-positive CTCs before treatment. Log-Rank Test WW =  − 0.3029, Sum = 12.674, Var = 2.7558, Test statistic =  − 0.182442, *p* = 0.85524

When analyzing the disease-free and overall 5-year survival of patients with breast cancer, depending on the presence of CTCs before the start of special treatment, no statistically significant differences were obtained (Figs. 1, 2.)

**Fig. 3:**
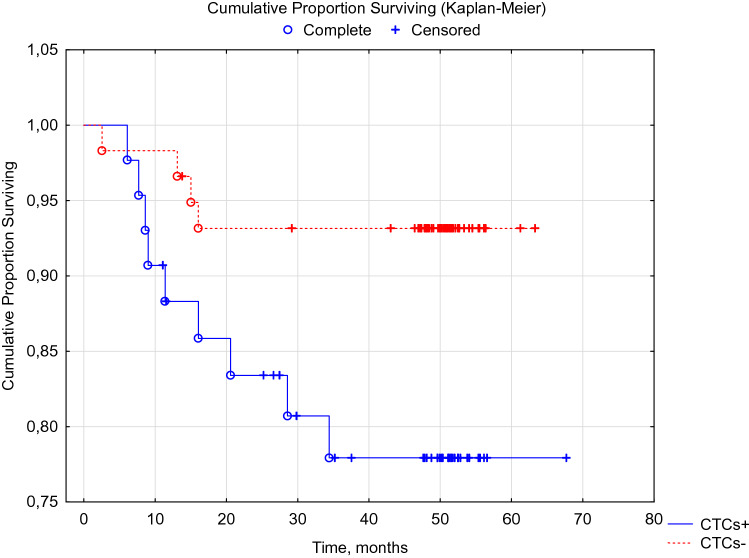
DFS of breast cancer patients with preservation of mRNA BIRC5- and HER2-neu-positive CTCs after a complex of special antitumor treatment. Log-Rank Test WW = 3.8374, Sum = 12.839, Var = 3.1617, Test statistic = 2.158130, *p* = 0.03092

**Fig. 4:**
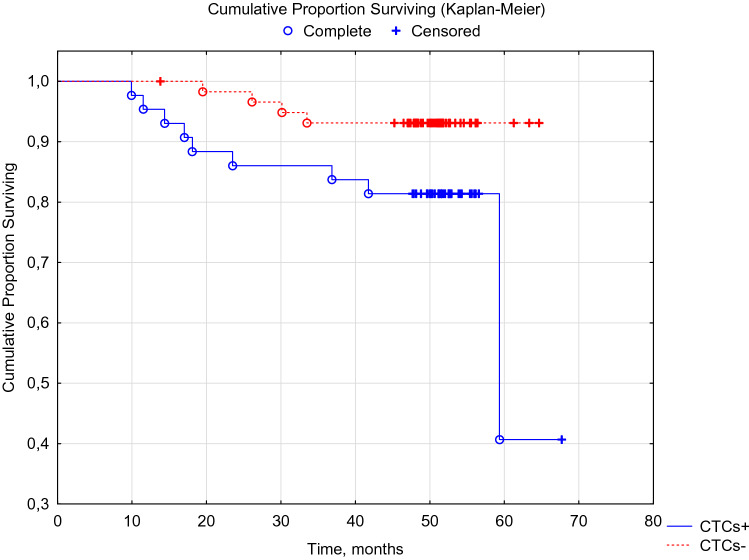
OS of patients with breast cancer with preservation of mRNA BIRC5- and HER2-neu-positive CTCs after a complex of special antitumor treatment. Log-Rank Test (Sheet1 in total) WW = 3.7422, Sum = 12.674, Var = 3.1212, Test statistic = 2.118162, *p* = 0.03416

When analyzing recurrence-free and overall 5-year survival of patients with breast cancer, depending on the preservation of CTCs after completion of a complex of special antitumor treatment, statistically significant differences were obtained (Figs. 3, 4.)

When analyzing the risks of disease progression depending on the presence of mRNA BIRC5- and HER2-neu-positive CTCs depending on the stage of treatment, it was found that there is a statistically significant increase in the relative risk of disease recurrence as CTCs persist in the peripheral blood both after radical surgery and after completion of systemic therapy compared with the indicators obtained before the start of anticancer treatment (Table 4).

**Table 4:** Indicators of relative risks BC progression after treatment depending on the presence of mRNA BIRC5- and HER2-neu-positive CTCs before starting treatment and maintaining them after treatment

Risk indicators	Index		
	BIRC5 mRNA- and HER2-neu-positive CTCs before treatment	BIRC5 mRNA- and HER2-neu-positive CTCs after surgery (MRD)	BIRC5 mRNA- and HER2-neu-positive CTCs after treatment (MRD)
Absolute risk in the main group (EER)	0.31	0.53	0.55
Control group absolute risk (CER)	0.02	0.01	0.010
Relative risk (RR)	14.71	49.77	52.36
Standard error of relative risk (S)	0.99	1.001	1.00
Lower bound 95% CI (CI)	2.08	6.99	7.36
Upper limit 95% CI (CI)	104.23	354.14	372.55
Reducing relative risk (RRR)	13.71	48.77	51.36
Risk difference (RD)	0.29	0.52	0.54
Number of patients to be treated (NNT)	3.43	1.93	1.87
Sensitivity (Se)	0.97	0.97	0.97
Specificity (Sp)	0.37	0.74	0.76

Analysis of OS and DFS, as well as risk analysis, led to the conclusion that the retention of CTCs in the peripheral blood of patients after the completion of special treatment significantly reduces the parameters of both OS and DFS. Therefore, it is logical to achieve maximum CTCs eradication by prescribing adjuvant CTC-oriented therapy.

According to the current algorithm, all patients received systemic therapy, depending on the molecular biological subtype of the tumor, the stage of the tumor process.

Analysis of dynamic monitoring of CTCs expressing the BIRC5 and HER2-neu genes during direct drug treatment in relation to the frequency of CTC detection after surgery, a significant decrease in the frequency of identification of targeted CTCs can be achieved only by the sequential administration of anthracyclines and taxanes (paclitaxel) AC-T, which allows to reduce the incidence of MRD in comparison with other schemes from 52.6 to 15.8% (*p* = 0.006). The most commonly used CAF therapy regimen was effective in eradication of CTCs expressing the BIRC5 and HER2-neu genes, which was comparable to the results of the AS therapy (*p* > 0.05). The combined use of the targeted monoclonal antibody trastuzumab in conjunction with paclitaxel (T-Trust regimen) in the presence of overexpression of the HER2-neu oncoprotein in tumor tissue made it possible to reduce the detection rate of targeted CTCs by 6.6%, but this decrease is not statistically significant (*p* > 0.05). Hormone therapy also did not lead to a significant decrease in CTCs expressing genes for the antiapoptotic protein survivin and the epidermal growth factor receptor (*p* > 0.05). A summary of the efficacy of various systemic adjuvant therapy regimens for CTC eradication is presented in Table 5.

**Table 5:** Effectiveness of various schemes systemic adjuvant therapy for breast cancer for the eradication of BIRC5 mRNA- and mRNA HER2-neu-positive CTCs

Systemic therapy scheme	Number of patients	CTC-positive cases						*p*
		Before surgery		After surgery		After systemic therapy		
		Abs. value	%	Abs. value	%	Abs. value	%	
AC	21	15	71.4	10	47.6	11	52.4	0.971
CAF	23	17	73.9	11	47.8	13	56.5	0.983
AC–T	19	16	84.2	10	52.6	3	15.8	0.006
T–trast	15	10	66.7	5	33.3	4	26.7	0.075
Hormone therapy	24	11	45.8	9	37.5	10	41.7	0.737
Weighty	102	69	67.6	45	44.1	41	40.2	0.130

Based on the results of the first stage study, it can be concluded that maintaining CTCs during therapy, as well as after its completion, significantly reduces the rates of both relapse-free and overall survival of patients with non-metastatic breast cancer. This may result in the conclusion that it is necessary to strive to achieve maximum eradication of CTCs in the peripheral blood, taking into account the use of the most effective schemes of adjuvant systemic therapy.

The second stage of the study is a prospective randomized controlled study (*n* = 64 tested group, *n* = 64 control group) optimization of adjuvant therapy in patients with non-metastatic breast cancer taking into account CTCs with an assessment of the effectiveness of the standard therapy regimen and the optimized therapy regimen with an assessment of tumor-specific overall and disease-free survival.

After randomization using a random number generator, 128 people were initially divided into 2 groups of 64 people, control and test, respectively. Both groups were tested for the presence of CTCs in the peripheral blood before treatment, after surgery, and after every two courses of systemic therapy. The control group received a standard treatment regimen, taking into account the stage of the tumor process and the molecular biological subtype of the tumor. The test group initially received therapy in accordance with the stage of the tumor process and the molecular biological subtype of the tumor; however, in the presence of CTCs in the peripheral blood, after 2 courses of systemic therapy, therapy was changed, in particular, anthracycline-containing regimens (CAF, AC) to taxanes, in particular paclitexel (T); four courses were carried out, and in the presence of CTCs in the peripheral blood, the therapy was changed to gemcitabine and 4 weekly injections of the drug were carried out with an assessment of MRD.

The adaptive randomization model implied that if patients in the control group appeared in whom CTCs were not initially diagnosed in the process, but began to emerge in the process of adjuvant therapy, then they should be transferred to the test group. In the process of adaptive randomization after 2 courses of anthracycline-containing regimens, 4 cases of newly diagnosed CTCs in the peripheral blood were found in the control group, which, taking into account the adaptive model of maximum utility (Maximum Utility Model), were transferred to the test group and the treatment regimen was changed.

So as a result, the ratio of the control and test group was 60 to 68 cases.

When analyzing the state of the peripheral blood of this subgroup of patients, it was found that 39 out of 64 (60.9%) had CTCs that expressed the BIRC5 and HER2-neu genes.

After radical surgical interventions, targeted CTCs—and, accordingly, MRD was diagnosed—were identified in 27 patients, which amounted to 42.1%.

In accordance with the data obtained after surgical treatment, namely information about the pathological stage of the tumor process, the molecular biological subtype of the tumor, further adjuvant treatment of the tested subgroup of patients was planned. Based on the criteria of risk categories, for the establishment of which it is necessary to use the recommendations of the International Consensus on the treatment of primary breast cancer, 47 patients (73.4%) were assigned to the groups of intermediate and high risk of disease recurrence, and, accordingly, chemotherapy was indicated for them in an adjuvant mode.

17 women (26.6%) received adjuvant hormone therapy with tamoxifen after radical Madden mastectomy or after RT, carried out as a combination therapy after radical breast resection.

After 2 courses of adjuvant PCT, as well as after 2 months of taking tamoxifen (alone or after RT), MRD was diagnosed by searching for CTCs expressing the BIRC5 and HER2-neu genes. It was found that 17 patients (26.6%), referred to the groups of intermediate and high risk of disease recurrence, who received chemotherapy in an adjuvant mode according to one of the regimens (CAF, AC or AC—T), had MRD confirmation.

It should be noted that in the control group after 2 courses (CAF, AC), 4 CTC-positive cases were found, which were initially CTC negative. They were transferred to the test group according to the adaptive randomization model.

Thus, the total incidence of MRD in the tested group after 2 courses of APCT was 21 out of 68 patients (30.9%).

According to the developed method, these 21 women underwent a change in therapy to taxanes, in particular, to paclitaxel. After 2 courses of paclitaxel, MRD was also diagnosed. A decrease in the incidence of MRD was noted to 15 cases. Thus, it was possible to achieve eradication of targeted CTCs in 6 patients.

After two more courses of monochemotherapy with paclitaxel (4 courses in total), it was found that targeted CTCs, expressing both the BIRC5 gene and the HER2-neu gene, remained in the peripheral blood of 12 patients. Thus, MRD was diagnosed in 12 patients (17.6%) of the tested subgroup.

This fact may indicate the formation of a resistant clone of tumor cells to taxanes.

12 patients who, after completing the entire complex of special treatment for breast cancer, retained BIRC5 mRNA- and HER2-neu mRNA-positive CTCs, for the eradication of the latter, 4 more weekly courses of monochemotherapy with gemcitabine in the mode of 800–1000 mg/m^2^ intravenously were carried out. After 4 courses of monochemotherapy with gemcitabine, no patient had targeted CTCs in the peripheral blood. Thus, 100% eradication of tumor cells in the peripheral blood was achieved.

The dynamics of CTC determination in the control group receiving standard therapy was as follows. Before surgery, the incidence of CTC-positive cases was 37 out of 64 (57.8%). After radical surgery—25 out of 64 (39.0%). After 2 courses of systemic therapy, 25 out of 64 patients were also diagnosed with MRD; however, in 21 women, CTCs remained from the baseline, and in 4 women CTCs were diagnosed for the first time and they were transferred to the test group. Accordingly, the incidence of MRD in the control group was 21 out of 60 (35.0%). After completion of all courses of systemic therapy, the frequency of CTC-positive cases was 20 out of 60, which was 33.3%.

**Fig. 5:**
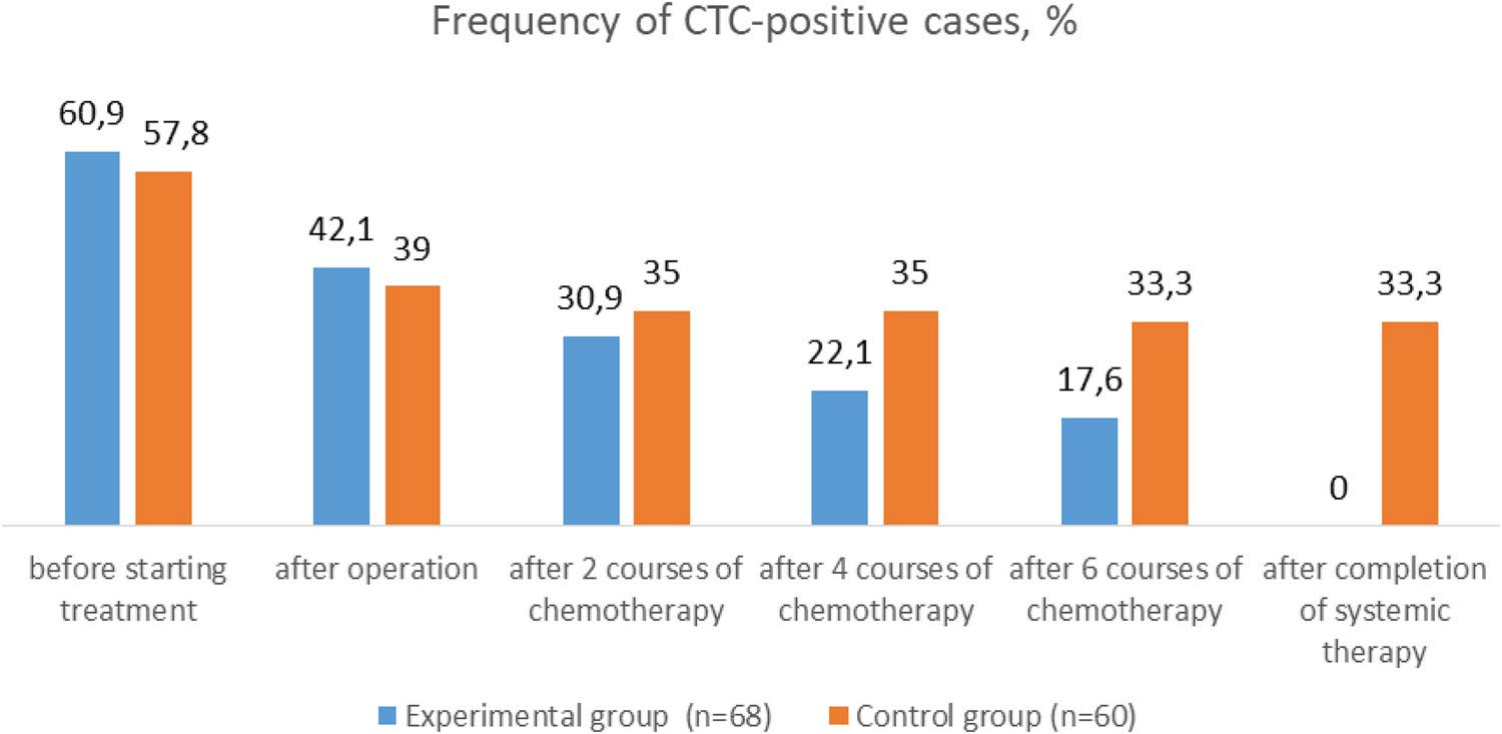
Frequency of CTC-positive cases in the experimental and control groups

The summary data on the dynamics of changes in the CTC status of patients in the control and test groups are presented in Fig. 5.

**Fig. 6:**
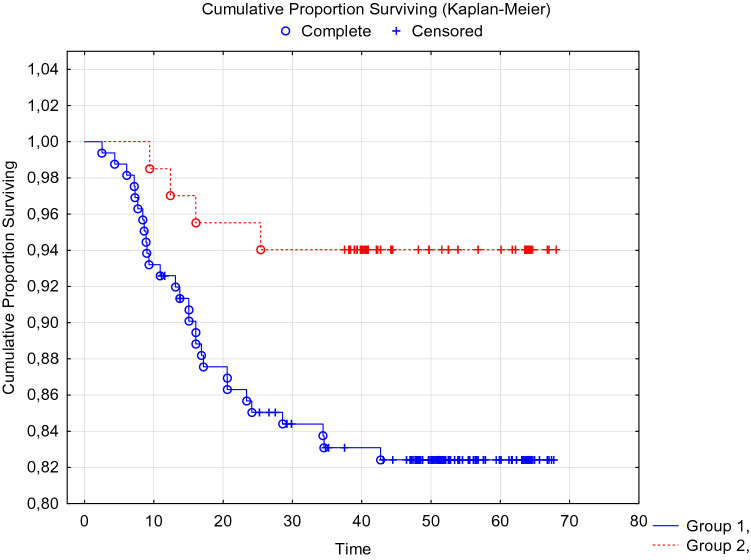
DFS of breast cancer patients in the experimental (red marker) and control (blue marker) groups. Log-Rank Test WW = 5.8143, Sum = 31.809, Var = 6.6125, Test statistic = 2.261078, p = 0.02375

**Fig. 7:**
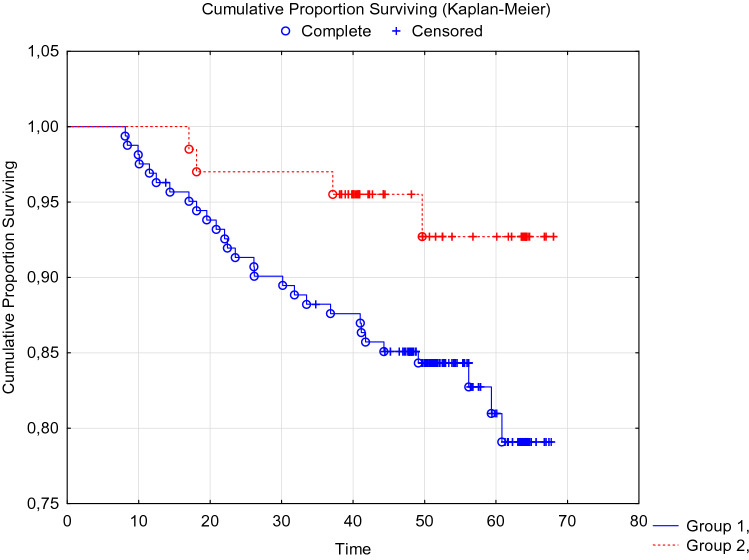
OS of patients with breast cancer in the experimental (red marker) and control (blue marker) groups. Log-Rank Test WW = 5.3888, Sum = 31.798, Var = 6.6103, Test statistic = 2.095965, p = 0.03609

In the adjuvant therapy optimization group taking into account CTCs (*n* = 68), the overall 5-year tumor-specific survival rate was 90.3 ± 3.8%, which was significantly higher than the indicator in the control group 78.7 ± 3.9% (p Log-Rank = 0.036). Disease-free tumor-specific survival in the experimental group was 88.0 ± 4.4%, which was significantly higher than the indicators of the control group 80.6 ± 3.3% (p Log-Rank = 0.023). Data are summarized in Tables 6 and 7 and Figs. 6 and 7.

**Table 6:** Disease-free survival of patients in the control and experimental groups of the study

Disease-free survival	Survival rates, % ± SE		
	1 year old	3 year old	5 year old
Control group (*n* = 60)	94.5 ± 2.1	83.1 ± 2.9	80.6 ± 3.3
Experimental group (*n* = 68)	97.7 ± 1.8	91.9 ± 3.3	88.0 ± 4.4

**Table 7:** Overall survival of patients in the control and experimental groups of the study

Overall survival	Survival rates, % ± SE		
	1 year old	3 year old	5 year old
Control group (*n* = 60)	96.6 ± 1.4	87.3 ± 1.6	78.7 ± 3.9
Experimental group (*n* = 68)	98.5 ± 1.5	93.47 ± 3.0	90.3 ± 3.8

## Discussion

The study of CTCs opens up entirely new horizons in terms of diagnosis and therapeutic targeting in relation to breast cancer [4–6].

The study of metastatic breast cancer has shed light on many aspects of tumor resistance when exposed to various chemotherapeutic agents. The findings are very valuable and should be extrapolated to CTCs.

For example, anthracyclines (inhibitors of topoisomerase II) disrupt the processes of replication and repair of deoxyribonucleic acid (DNA), which leads to apoptosis of tumor cells mediated by the p53 tumor suppression gene and caspase mechanisms. In addition, anthracyclines induce apoptosis by activating oxidative processes and generating free radicals.

Taxanes ("mitotic poisons") reversibly bind to the beta-subunit of tubulin, the dimers of which form microtubules, and block depolymerization [7].

As a result, an excess of defective microtubules is formed, which leads to the arrest of mitosis, followed by arrest of the cell cycle and apoptosis of the tumor cells [8].

Proteins of the ABC family adenosine triphosphate-binding cassette (ABC) play an important role in the development of tumor chemoresistance [9]: P-glycoprotein (P-gp), encoded by the MDR1 gene; multidrug-resistance-associated protein 1 (MRP1); breast cancer resistance protein (BRCP), encoded by the MXR gene [10, 11].

The main representative of the ABC family, P-glycoprotein, carries out ATP-dependent transport of toxic substances, including cytostatics and their metabolites, across the cell membrane. Overexpression of the P-glycoprotein (or the MDR1 gene) leads to increased excretion of the drug from the cell using the P-glycoprotein pump and a decrease in the concentration of the cytostatic in the cell itself, providing resistance to both anthracyclines and taxanes [12–14].

Anthracycline resistance may also be associated with overexpression of BCRP, activation of antioxidant defense mechanisms, topoisomerase II mutations, as well as overexpression of transcription-related DNA repair mechanisms or impaired apoptotic signaling pathway [15–19].

Taxanes bind to the beta-subunit of microtubulin, therefore resistance to them can be mediated by mutations of the beta-tubulin gene or overexpression of beta-tubulin type III, as well as overexpression of microtubule-associated proteins, such as survivin (BIRC5) or changes in staged mitotic signaling proteins [20–23].

With the development of resistance to chemotherapy drugs of basic breast cancer therapy, which is associated with an increase in the expression of genes of the ABC transporter family [24], drugs belonging to the group of antimetabolites, a subgroup of pyrimidine antagonists, may be effective. The most common drugs in our republic are gemcitabine and capecitabine.

Gemcitabine, an antimetabolite converted to difluorodioxycytidine triphosphate, inhibits DNA synthesis by inhibiting DNA polymerase, resulting in termination of DNA strand elongation. Diphosphate inhibits the activity of ribonucleotide reductase, thereby depleting intracellular stores of dioxyuridine monophosphate, which is required for DNA synthesis [25]. According to available data, gemcitabine has a pronounced antitumor activity and good tolerability in various malignant neoplasms, including breast cancer.

Monotherapy with gemcitabine resulted in responses to treatment in 37% of cases in the first line of chemotherapy [26], in 26% of cases in the second line [27], and in 18% of cases in the third line [28]. Gemcitabine has a unique mechanism of action and a favorable toxicity profile, thereby limiting the risk of developing resistance and excessive toxicity in pre-treated patients, making it an excellent agent for combination chemotherapy. The results of recent phase II and III studies examining combinations of gemcitabine with taxanes, platinum-containing drugs, vinorelbine, anthracyclines, and 5-fluorouracil showed advantages of using combinations with gemcitabine compared to using any of the listed drugs in monotherapy, especially in previously treated patients … The efficacy of gemcitabine monotherapy has been studied in at least six phase II clinical trials [26–31]. First-line response rates were 37% and 14% [26, 28]. In the remaining studies, conducted in most cases among previously treated patients, the response rate reached 17–29% [27–29, 31].

The results obtained from a multistage, controlled, randomized trial confirm the clinical significance of CTCs as a predictor and predictor of therapy.

## Conclusions

The use of the method of treatment of CTC-oriented personalized adjuvant therapy for non-metastatic breast cancer, based on the timely transition from an ineffective adjuvant PCT regimen to taxanes (without waiting for the completion of all planned courses), as well as additional monochemotherapy with gemcitabine (if necessary) can achieve 100% eradication of BIRC5 mRNA and HER2-neu mRNA of positive CTCs in the peripheral blood, which makes it possible to reliably increase recurrence-free 5-year survival by 7.4% (*p* = 0.023) to 88.0 ± 4.0 and overall 5-year survival by 11.6% (*p* = 0.036) to 90.3 ± 3.8%.
